# Supplementary material for: Developing a protocol for adapting multimedia patient-reported outcomes measures for low literacy patients
Source: PLoS One. 2021 Jun 4;16(6):e0252684. doi: 10.1371/journal.pone.0252684 (PMC8177453; doi:10.1371/journal.pone.0252684)
Supplement: Attachment — Submitted filename: Response to Reviewers.docx [file pone.0252684.s001.docx]

Dear PLOS ONE,

Thank you for the opportunity to revise our manuscript titled, “Developing a Protocol for Adapting Multimedia Patient-Reported Outcomes Measures for Low Literacy Patients.” Thank you to the reviewers for their thoughtful comments; we believe that our manuscript is substantially improved thanks to their feedback. The remainder of this letter includes a Table of Revisions, in which we provide our response as well as the corresponding changes to our manuscript. Of note, the line numbers cited in the last column, “Manuscript Revisions,” correspond to those in the document, “Revised Manuscript with Track Changes.”

| **Reviewer Comment** | **Response** | **Manuscript Revisions** |
| --- | --- | --- |
| *Journal Requirements* | | |
| “1. Please ensure that your manuscript meets PLOS ONE's style requirements, including those for file naming.” | The style requirements have been reviewed. | Several formatting changes have been made in the file names, title page, abstract, manuscript body, and references to align with journal requirements. |
| “2. We note that you have stated that you will provide repository information for your data at acceptance. Should your manuscript be accepted for publication, we will hold it until you provide the relevant accession numbers or DOIs necessary to access your data. If you wish to make changes to your Data Availability statement, please describe these changes in your cover letter and we will update your Data Availability statement to reflect the information you provide.” | We have made changes to our data availability statement. | This Registered Report Protocol does not include pilot data; there is therefore no data to be made available at this time. Details of these changes are also provided in the cover letter, as requested. |
| “Thank you for including your ethics statement on the online submission form:  "This study has been approved by the MedStar Health Research Institute institutional review board. The ID is STUDY00002319".   To help ensure that the wording of your manuscript is suitable for publication, would you please also add this statement at the beginning of the Methods section of your manuscript file.” | This has been done as requested. | Line 168: Added sub header, “Research ethics.”  Lines 169-170: Added the sentence, “This study has been approved by the MedStar Health Research Institute institutional review board. The identification number is STUDY00002319.” |
| *Reviewer #1* | | |
| “The Multimedia Adaptation Protocol (MAP) described in this manuscript is innovative and has the potential to improve collection of PROMs in diverse patient populations. It would be helpful to more clearly separate issues of individual question understandability from issues of self-administration.” | Thank you for your supportive comments. We agree that the manuscript could benefit from addressing these two separate issues more clearly. | Line 118-120: Added the sentence, “Text-based PROMs require basic literacy skills for question comprehension, which in turn precludes illiterate or very limited literacy patients from accurate self-administration.” |
| “Future sustainability and costs of any multimedia solutions should be briefly discussed.” | We agree with this suggestion. | Line 366-367: Added the sentence, “Further research on cost-effectiveness is also needed to evaluate the sustainability of developing mPROMs and administering them on a large scale.” |
| “- Lines 59-60: This phrase is unclear: “...those that incorporate visual elements still rely on text to convey meaning.” How do visual elements rely on text to convey meaning?” | We agree that this phrase is confusing and have revised it. | Line 117-118: This phrase in question was revised to state, “Although there have been efforts to simplify PROMs, the majority still rely on text elements to convey meaning or question details.” |
| “- Line 61: This phrase (“patients who are illiterate or have limited literacy may still not be able to complete them”) should be clarified. Do the authors mean that they may not be able self-administer questionnaires if they are only text-based? Interviewer-administration has often been used to help low literacy patients with self-report. It would be helpful to clarify mode (self-administration vs. interviewer-administration) and method (paper, computer, telephone) throughout this manuscript.” | We agree that these distinct concepts require clarification. | Line 79-80: “Administration” has been clarified to “Self-administration of patient-reported outcomes measures (PROMs) by patients with low literacy is a methodologic and implementation challenge.”  Line 118-120: This sentence has been rewritten to state, “Text-based PROMs require basic literacy skills for question comprehension, which in turn precludes illiterate or very limited literacy patients from accurate self-administration.”  Line 131-133: This sentence has been rewritten to state, “While interviewer-administration by clinical or research staff is an alternative mode of administration that allows low literacy patients to self-report, it is labor-intensive and expensive.”  Line 133: “Administration by staff” was edited to “interviewer-administration” to improve word choice consistency.  Line 153-154: Added clarification that multimedia PROMs are an alternative method to administering PROMs; now states, “"Talking Touchscreen” (TT), initially developed for low literacy cancer patients, is an alternative method for administering multimedia PROMs (mPROMs) with audiovisual components.”  Line 336-338: Revised sentence to state, “This intrinsically limits accurate measurement of PROs in low and limited literacy populations when those PROs are obtained via self-administration.” |
| “- Line 86: The TT is an mPROM method of administration; the method itself does not have “good internal consistency” which is an attribute applicable only to multi-item questionnaires.” | We agree. | Line 152-154: Added an additional sentence to clarify TT as a method for administration: “PROMs are routinely administered via two methods: paper-and-pencil or electronically. “Talking Touchscreen” (TT), initially developed for low literacy cancer patients, is an alternative method for administering multimedia PROMs (mPROMs) with audiovisual components.”  Line 156-158: Revised to state, “Questionnaires administered using TT as the method of administration have good internal consistency, demonstrating the potential for mPROMs to improve accessibility of PROs in low literacy populations.” |
| “Lines 160-162: Observation of patients, caregivers, and staff will be helpful to “facilitate understanding the processes of PROM completion and identification of challenges and opportunities in these processes.”” | Thank you. |  |
| Lines 164-166: The two types of interviews (cognitive and discovery) will be very useful “to understand how patients interpret and respond to each item in the source instrument” and “elicit a discussion of ease of use, areas of poor design, and perceived barriers and strategies for completion.” | Thank you. |  |
| Line 175: The technological terms used here (“low-fidelity wireframes or application interfaces”) may not be familiar to most readers. | We agree that we should avoid this technical jargon. | Line 289: Revised to, “the prototypes can be static visual representations”  Line 301: Revised to, “mPROM version 2.0 can be upgraded to a functional and interactive prototype…” |
| Lines 179-180: It is not clear how “text back-adaptations” will be “compared to the source instrument to assess for conceptual equivalence.” Conceptual equivalence should also be defined. | We agree that this requires clarification. | Line 293-295: Sentence revised to, “These text back-adaptations are compared to the source instrument; this is done by assessing for conceptual equivalence, defined as the adapted instrument conveying the same content as the source instrument.” |
| *Reviewer #2* | | |
| “This paper presents a protocol (Multimedia Adaptation Protocol) for the adaptation of validated patient reported outcomes into a multimedia format to optimize administration for patients with limited health literacy. This paper could be used by other researchers as a template to help decrease disparities in research and I believe would have interest to PLOS ONE readers.” | Thank you for your supportive comments. |  |
| “Line 99: It would be helpful to describe the 3 states of the IQOLA, since the mPROM protocol is only using one of the stages.” | We agree. | Line 186-188: Added, “IQOLA developed a three-stage process that includes 1) translation, 2) psychometric testing of scaling assumptions, and 3) validating and norming of translations.” |
| “Line 111: how many experts were involved. 1 of each qual, social designers etc? How large was the development team?” | We have included additional information regarding size of the team. | Line 201-202: Revised to read, “…including two PRO experts, two qualitative research scientists, two social designers, three clinicians, and one biostatistician.” |
| “Figure 1: On my version I could not find a footnote. There are many acronyms that should be defined in a footnote. It was also hard to know from the figure if all of the 3 columns to the left informed the 4th MAP column and the MAP column is what researchers should do, or if researchers should engage in activities in all 4 columns? Line 132 says it incorporated HCD (pink) into the MAP, but the MAP is the final column. As above, it was hard to know whether column 4 is the final protocol and includes HCD or whether HCD is a separate step, as were the other columns.” | We have added a footnote for the acronyms.  The MAP includes the stages, HCD phases, and methods; researchers should engage in all 3. We agree that this was not clearly communicated and have edited the figure and text to clarify. | Figure 1 changes: To better illustrate that IQOLA’s translation protocol was used as a guiding conceptual framework that informed the MAP, IQOLA’s translation protocol is now in grey and written vertically on the left of the figure. To better illustrate that the remaining three columns are all part of the MAP, the headers were revised so that there is one main header, “Multimedia Adaptation Protocol” that spans the three subheaders (“stages,” “human-centered design phases,” “methods”) which remain in color.  Line 208: Revised the figure legend to read, “The IQOLA Project Translation Protocol was used as a guiding conceptual framework to develop the MAP. The parallel components of the MAP include four stages (green), a human-centered design approach (red), and eight distinct methods (blue).”  Lines 218-220: Added to figure legend, “IQOLA = International Quality of Life Assessment; MAP = Multimedia Adaptation Protocol; HCD = human-centered design; mPROM = multimedia patient-reported outcomes measure; v1.0 = version 1.0; v2.0 = version 2.0; X.0 = final version”  Lines 228-230: Revised to state, “The phases of HCD (empathize, define, ideate, prototype, test) are how the MAP stages are operationalized (pink, Fig 1).”  Lines 231-235: Added a sentence to clarify how the MAP stages, HCD phases, and methods relate to each other: “The MAP is represented as three columns in Fig 1 because of the parallel nature of the MAP stages, HCD phases, and methods. For example, reading the MAP horizontally, the MAP forward adaptation stage begins with the HCD phase of empathizing that is carried out with observation and discovery and cognitive interviews.” |
| “Line 137: How large was this board and are any demographic characteristics available? How many iterations of the MAP did they review?” | We agree that additional detail about the board would be helpful. The board’s specific demographics unfortunately are not made available to us. | Line 238-240: Revised to state, “Consultation from this eight-member board, which comprised a racially and ethnically diverse group of four community members as well as four clinical research experts, aided in the refinement of protocol elements.”  Line 240-241: Added, “This board reviewed one iteration of the MAP.” |
| “Starting at Line 158 to describe the MAP methods over several paragraphs and the Figure: I think it would help to label the steps listed in this paragraph and have them linked to the box you are referring to in the Figure. As above, I had a hard time understanding how to read and use the figure to adapt PROM. The authors could consider numbering or using letters in the text that then correspond to letters/numbers on the figure so it would be easier to follow the step by step approach to this development.” | We agree that it may be hard to follow the figure. We have taken this reviewer’s suggestion of numbering certain steps to correspond to the text. | Line 268: Revised subheader to, “Multimedia Adaptation Protocol (MAP) methods”  Line 269: Added, “The protocol’s methods include eight distinct steps (blue, Fig 1).”  Figure 1: Numbered the steps of the MAP methods: (1) observation; (2) discovery and cognitive interviews; (3) analysis; (4) ideation workshop and cycles of prototyping and refinement; (5) researcher and user testing for conceptual equivalence; (6) consultation with community advisory board; (7) interviews; (8) psychometric evaluation.  Lines 269-333: Numbering has been added to the manuscript text, such that the numbers in the text correspond to the numbers in Fig 1 in a step-by-step fashion. |
| “As this is a methods paper and it should be able to be replicated by these and other researchers, I would have also found a table or a figure with a step by step approach as if not more helpful that the conceptual framework (which as above was still not clear to me what parts were used when and how the IQOLA and HCD were included in the MAP. It was hard to match the text up with the figure.” | We agree that this can be improved with a step-by-step approach that is highlighted in both the text and figure. | Figure 1: To deemphasize the conceptual framework and to clarify that it is not part of the MAP, IQOLA’s translation protocol is now in grey and written vertically to the figure’s left.  Figure 1: Numbered the steps of the MAP methods: (1) observation; (2) discovery and cognitive interviews; (3) analysis; (4) ideation workshop and cycles of prototyping and refinement; (5) researcher and user testing for conceptual equivalence; (6) consultation with community advisory board; (7) interviews; (8) psychometric evaluation.  Lines 269-333: Numbering has been added to the manuscript text, such that the numbers in the text correspond to the numbers in Fig 1 in a step-by-step fashion. |
| “Line 183: “Identify the prototype that performs the best.” Can the authors describe what that means? I did not see any mention of psychometric properties of the tool. What psychometric properties will be measured and will they define performance?” | We agree that this is confusing. | Upon re-evaluation, this sentence is redundant and has been deleted. This sentence is redundant because prototypes will be assessed and chosen based on conceptual equivalence, which is explained by the prior sentence in Lines 295-296 (“Prototypes that demonstrate poor conceptual equivalence relative to the others will be discarded.”) |

Thank you sincerely for your consideration of our revised manuscript.

Sincerely,

Aviram M. Giladi, MD, MS
