# Supplementary material for: Developing a protocol for adapting multimedia patient-reported outcomes measures for low literacy patients
Source: PLoS One. 2021 Jun 4;16(6):e0252684. doi: 10.1371/journal.pone.0252684 (PMC8177453; doi:10.1371/journal.pone.0252684)
Supplement: Attachment — Submitted filename: Response to Reviewers.docx [file pone.0252684.s002.docx]

Dear PLOS ONE,

Thank you for the opportunity to revise our manuscript titled, “Developing a Protocol for Adapting Multimedia Patient-Reported Outcomes Measures for Low Literacy Patients.” Thank you to the reviewers for their thoughtful comments; we believe that our manuscript is improved thanks to their feedback. The remainder of this letter includes a Table of Revisions, in which we provide our response as well as the corresponding changes to our manuscript. Of note, the line numbers cited in the last column, “Manuscript Revisions,” correspond to those in the document, “Revised Manuscript with Track Changes.”

| **Reviewer Comment** | **Response** | **Manuscript Revisions** |
| --- | --- | --- |
| *Journal Requirements* | | |
| “Please review your reference list to ensure that it is complete and correct. If you have cited papers that have been retracted, please include the rationale for doing so in the manuscript text, or remove these references and replace them with relevant current references. Any changes to the reference list should be mentioned in the rebuttal letter that accompanies your revised manuscript. If you need to cite a retracted article, indicate the article’s retracted status in the References list and also include a citation and full reference for the retraction notice.” | The reference list has been reviewed. It is complete and correct. We did not identify any cited papers that have been retracted. | No changes necessary. |
| *Reviewer #1* | | |
| “This is a revised manuscript describing the Multimedia Adaptation Protocol. The revision satisfactorily addressed most, but not all, of the previous review. In addition, the Line numbers mentioned in the authors’ response do not seem to match the manuscript.” | Thank you for your supportive comments. Our apologies for mismatched line numbers. | None |
| “This statement should be revised: “Questionnaires administered using TT as the method of administration have good internal consistency…” Internal consistency reliability is a property of a particular questionnaire. It is not accurate to imply that all questionnaires have demonstrated internal consistency reliability, unless the authors have done a comprehensive review of every study that used the TT.” | Thank you for this comment. We agree. | Lines 78-81: Sentence revised to, “The Functional Assessment of Cancer Therapy-General (FACT-G) and the Short Form-36 Health Survey (SF-36), when administered using TT, demonstrated good internal consistency; this supports the potential for mPROMs to improve accessibility of PROs in low literacy populations.” |
| “The revised manuscript still uses jargon that may not be familiar to most readers, e.g., “a functional and interactive prototype.”” | Thank you for this comment. We agree. | Line 199: Revised to, “…a working prototype…” |
| *Reviewer #2* | | |
| “The authors have done a nice job responding appropriately to all of my and other reviewer comments. This version is much clearer. I appreciate the changes to the text that help match the description to the figure and the simplified figure. I have nothing more to add.” | Thank you. | None |

Thank you sincerely for your consideration of our revised manuscript.

Sincerely,

Aviram M. Giladi, MD, MS
